# Advanced imaging shows extra-articular abscesses in two out of three adult patients with septic arthritis of the native hip joint

**DOI:** 10.5194/jbji-9-27-2024

**Published:** 2024-02-02

**Authors:** Jordi Cools, Stijn Ghijselings, Fred Ruythooren, Sander Jentjens, Nathalie Noppe, Willem-Jan Metsemakers, Georges Vles

**Affiliations:** 1 Department of Orthopaedic Surgery, University Hospitals Leuven – Gasthuisberg, Leuven, Belgium; 2 Institute for Orthopaedic Research and Training (IORT), Catholic University Leuven, Leuven, Belgium; 3 Department of Nuclear Medicine, University Hospitals Leuven – Gasthuisberg, Leuven, Belgium; 4 Department of Radiology, University Hospitals Leuven – Gasthuisberg, Leuven, Belgium; 5 Department of Trauma Surgery, University Hospitals Leuven – Gasthuisberg, Leuven, Belgium; 6 Department of Development and Regeneration, KU Leuven, Leuven, Belgium

## Abstract

**Background**: Septic arthritis (SA) of the native adult hip is a rare orthopaedic emergency requiring prompt diagnosis and treatment. As clinical presentation and laboratory findings are frequently atypical, advanced imaging is often requested. This retrospective study aimed to investigate the prevalence and pattern of extra-articular infectious manifestations and their implications for pre-operative advanced imaging in patients with proven SA of the native hip joint. **Methods**: Out of 41 patients treated surgically for SA of the native hip during a 16-year period at our tertiary referral hospital, 25 received advanced imaging (computed tomography (CT), magnetic resonance imaging (MRI), or fluorodeoxyglucose positron emission tomography (FDG PET-CT)) prior to initial intervention. For each investigation, a specific set of variables was systematically interpreted, and the most suitable surgical approach was determined. The prognostic value was evaluated by comparing specific outcome measures and the extent of extra-articular involvement. **Results**: It was found that 32 % of patients had an abscess in one anatomical region, 32 % of patients had abscesses in multiple anatomical regions, and only 36 % of patients had no substantial abscess. Gluteal abscesses were especially common in patients with SA due to contiguous spread. Abscesses in the iliopsoas region were more common in patients with SA due to hematogenous seeding. A combination of several different surgical approaches was deemed necessary to adequately deal with the various presentations. No significant prognostic factors could be identified. **Conclusion**: We recommend performing advanced imaging in patients with suspected or proven septic arthritis of the native hip joint, as extra-articular abscesses are present in 64 % and might require varying anatomical approaches.

## 1 Introduction

Septic arthritis (SA) of the native adult hip is a rare orthopaedic emergency that requires prompt diagnosis and treatment (Balato et al., 2021; Lum et al., 2018; Carpenter et al., 2011; Clerc et al., 2011; Rutz and Brunner, 2009). The reported incidence of acute bacterial arthritis of a single native joint in developed countries fluctuates around 10 per 100 000 patients per year in the general adult population (Arieli et al., 2021; Kennedy et al., 2015; Clerc et al., 2011; Mathews et al., 2010; Margeretten et al., 2007; Kaandorp et al., 1995). The hip joint is involved in only a small fraction of these cases (Ross, 2017; Kennedy et al., 2015; Carpenter et al., 2011). Given this low incidence, data on the topic mainly originate from retrospective cohort studies (Mathews et al., 2010). As a result, there are no generally accepted diagnostic and therapeutic algorithms for SA of the native hip joint (Balato et al., 2021). Treatment of acute bacterial arthritis consists of surgical intervention and antibiotic therapy (Goldberg and Sexton, 2022; Balato et al., 2021; Lum et al., 2018; Ross, 2017; Mathews et al., 2010; Rutz and Brunner, 2009). Surgical modalities range from arthroscopic or open debridement to (staged) total hip arthroplasty (THA) and (modified) Girdlestone procedures, using a variety of anatomical approaches (Balato et al., 2021; D'Angelo et al., 2021; Russo et al., 2021; Davis and Zamora, 2020; Lum et al., 2018; Rutz and Brunner, 2009).

Clinical presentation and laboratory findings are frequently atypical and imprecise (Ross, 2017; Carpenter et al., 2011; Clerc et al., 2011; Margaretten et al., 2007). Fever at presentation is, for example, only present in around 60 % of cases (Ross, 2017; Clerc et al., 2011; Mathews et al., 2010; Margaretten et al., 2007), and joint pain might be confounded by pre-existing osteoarthritis (Clerc et al., 2011). In addition, serum markers, more than synovial fluid markers, suffer from low sensitivity and specificity (Varady et al., 2022; Ross, 2017; Carpenter et al., 2011).

It is apparent that both over- and underdiagnosing SA of the native hip joint will have severe consequences. Advanced imaging, for example, fluorodeoxyglucose positron emission tomography (FDG PET), magnetic resonance imaging (MRI), and computed tomography (CT), is therefore often requested in the diagnostic work-up of patients in whom clinical presentation and/or biochemical findings are ambiguous (Goldenberg and Sexton, 2022; Casali et al., 2021; Lum et al., 2018; Ross, 2017; Simpfendorfer, 2017; Mathews et al., 2010; Karchevsky et al., 2004; Weishaupt and Schweitzer, 2004; Learch and Farooki, 2000). It can demonstrate inflammation in and around the hip joint, although it is important to note that it is not always possible to differentiate between other non-infectious types of inflammation (Ravn et al., 2023; Casali et al., 2021; Mathews et al., 2010; Weishaupt and Schweitzer, 2004). It can also provide information on pre-existent osteoarthritis, avascular necrosis, and other joint pathologies that might indicate that joint sacrifice or replacement should be preferred over attempts at femoral head preservation (Davis and Zamora, 2020; Ross, 2017; Mathews et al., 2010). Additionally, FDG PET-CT can identify distant infectious foci (Casali et al., 2021).

The exact role of advanced imaging in the diagnostic work-up of patients with potential SA of the native hip joint remains unclear. Its use, to date, is mostly restricted to supporting or opposing diagnosis in patients with an ambiguous presentation (Goldenberg and Sexton, 2022; Casali et al., 2021; Davis and Zamora, 2020; Elsissy et al., 2020; Ross, 2017; Simpfendorfer, 2017). We hypothesised that the application might be broader since the presence of adjacent infections could directly influence surgical decision-making and outcomes (Rosenfeld et al., 2016). For example, Shoji et al. (2021) reported concomitant musculoskeletal infection in patients with an iliopsoas abscess as a risk factor for failure of conservative treatment.

Our primary aim was to determine the prevalence of extra-articular infectious manifestations on pre-operative advanced imaging in patients with proven SA of the native hip joint. Secondary aims were to determine whether a certain pattern of infectious manifestations could be identified, whether advanced imaging could guide surgical approach, and whether prognostic factors for prolonged hospital stay, number of surgeries, and femoral head preservation could be found.

## 2 Methodology

### 2.1 Study design

This retrospective study was approved by the medical ethics committee of University Hospitals Leuven, Belgium (S64930). The medical records of all patients with synovial fluid and/or tissue samples sent to the microbiology laboratory by orthopaedic surgeons between 1 January 2005 and 1 January 2021 were screened. All patients with a bacterial infection of the hip joint, confirmed on synovial fluid and/or tissue samples (see Definitions), were eligible for inclusion. Subjects with a history of arthroplasty in the affected joint were excluded. Each patient received a combination of surgical intervention (see Definitions) and targeted antibiotic treatment. At least one surgical intervention had to be performed at the University Hospitals Leuven. All patients meeting these requirements were subsequently screened for undergoing advanced imaging investigations prior to initial surgical intervention.

### 2.2 Definitions

Septic arthritis was defined as (1) the growth of microorganisms in either aerobic or anaerobic cultures from tissue or fluid samples obtained by arthrocentesis or arthrotomy and/or (2) synovial cell count exceeding 50 000 white blood cells per microlitre ( µ L), and >90% were polymorphonuclear neutrophils (PMNs) (Ravn et al., 2023; Ross, 2017; Margaretten et al., 2007). Route of infection was considered the result of contiguous spreading if the infection of the hip joint resulted from the dissemination of the pathogen through surrounding soft tissue (e.g. sacral decubital wounds). Direct inoculation infections resulted from iatrogenic introduction of the pathogen into the hip joint (e.g. intra-articular infiltrations). Hematogenous infections were the result of the pathogen being seeded into the hip joint from a distant source (e.g. endocarditis). We considered advanced imaging to be CT, MRI, and FDG PET-CT scans. Plain radiographs and ultrasound investigations were defined as basic imaging. Arthroscopic or open debridement, (staged) total hip arthroplasty (THA), and (modified) Girdlestone procedures were considered surgical interventions. A substantial abscess was defined as a collection >2 cm in diameter since iliopsoas abscesses (IPAs) with a transverse diameter ranging from 2 to 3 cm have been previously described to require (surgical) drainage (Shoji et al., 2021; Hsieh et al., 2013).

### 2.3 Data collection

Advanced imaging investigations were interpreted by an experienced nuclear physician (Sander Jentjens: FDG PET-CT) and an experienced musculoskeletal radiologist (Nathalie Noppe: CT and MRI), both blinded to clinical and biochemical information as well as the surgical outcome.

The following radiological variables were systematically collected for all patients: (1) localisation of the infectious manifestations: intra-capsular, intra-osseous involvement (osteomyelitis of the femoral head, femoral neck, greater trochanter, acetabulum or sciatic tuberosity), soft-tissue involvement of the iliopsoas region, gluteal region (gluteus maximus, medius and minimus muscle), external rotator region, adductor region, hamstring region, vastus lateralis region, and anterior region (tensor fascia latae (TFL) muscle, sartorius muscle and/or rectus femoris muscle) and (2) the presence/absence of abscesses. For patients who underwent FDG-PET imaging, additionally, the maximum standardised uptake value (SUVmax), the tumour-to-background ratio, and the presence of distant infectious foci were identified.

### 2.4 Surgical assessment

Advanced imaging investigations, including reports on the abovementioned data variables, were then made available to two orthopaedic hip surgeons (Georges Vles, Stijn Ghijselings). Blinded to the surgical interventions performed and the outcome, the most suitable anatomical approach for debridement was retrospectively determined for every case with an extra-articular collection present.

### 2.5 Statistical analysis

Statistical analysis was performed using IBM SPSS Statistics software for Macintosh version 28.0.1.1 (Armonk, NY). Proportion comparison between categorical variables was performed using Fisher's exact test, the Fisher–Freeman–Halton exact test, or the Chi-squared test depending on the number of observations. Comparison between continuous variables was carried out using Mann–Whitney U and Kruskal–Wallis tests (in the case of >2 independent variables). Statistical significance was set at p<0.05 . In case of multiple comparisons, a Bonferroni correction was applied.

**Table 1:** Advanced imaging modalities in the study population.

	Overall	Hematogenous	Contiguous	Direct
	N=41	seeding	spread	inoculation
		N=17	N=16	N=8
Advanced imaging	25 (60.98 %)	12 (70.59 %)	10 (62.50 %)	3 (37.50 %)
Number of investigations	0.9±0.86	1.12±0.93	0.88±0.81	0.5±0.76
FDG PET-CT	7	6	1	0
MRI	14	6	6	2
CT	16	7	7	2
CT + MRI	8	4	3	1
CT + FDG PET-CT	4	3	1	0

**Table 2:** Presence of an extra-articular abscess by route of infection and by imaging modality.

Extra-articular	Hematogenous seeding	Contiguous spread	Direct inoculation	p value
abscess	7/12 (58 %)	8/10 (80 %)	1/3 (33 %)	.214
>2 cm	MRI	CT	FDG PET-CT	p value
	10/14 (71 %)	11/16 (69 %)	3/7 (43 %)	.276

## 3 Results

### 3.1 Patient population

A sample of 41 patients with SA of the native hip joint was identified. Out of this population, 25 patients received at least one type of advanced imaging prior to initial debridement (mean age 57 years old, SD 21; male to female ratio ±3:1 ; mean Charlson Comorbidity Index (CCI) 3.4, SD 2.5; mean American Society of Anesthesiologists physical status score (ASA score) 2.6, SD 0.7; and median delay until initial debridement 12 d, interquartile range (IQR) 15) (Table 1). A total of 7 patients (4 male, 3 female) underwent FDG PET-CT, 14 patients (9 male, 5 female) received MRI, and CT was performed in 16 patients (12 male, 4 female). Only 1 patient underwent all three investigations, and 10 patients received two types of advanced imaging. In patients receiving multiple advanced imaging investigations prior to initial debridement, the study closest to the surgical intervention was used for between-patient comparison. In 12 cases, the SA was due to hematogenous seeding, in 10 due to contiguous spread, and in 3 due to direct inoculation. FDG PET-CT was predominantly performed in patients with SA due to hematogenous seeding. The other advanced imaging modalities (MRI and CT) were not significantly associated with a certain route of infection.

### 3.2 Prevalence of extra-articular infectious manifestations on pre-operative advanced imaging

**Figure 1:**
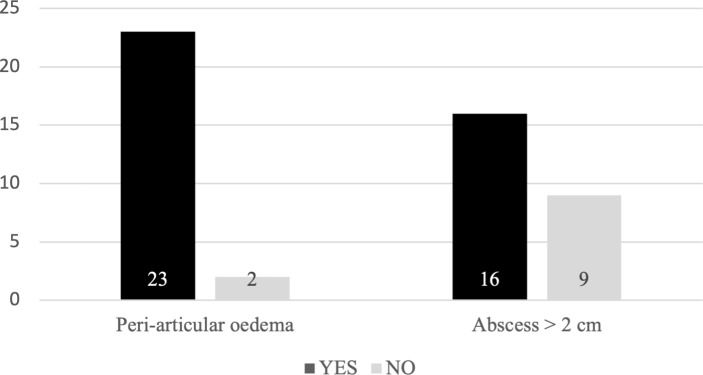
Extra-articular infectious soft-tissue manifestations.

Out of 25 patients, extra-articular manifestations of infection, ranging from small amounts of oedema to large abscesses, were observed in 23 patients (92 %). Excluding muscle oedema, at least one substantial abscess was observed in 16 patients (64 %) (Fig. 1). The prevalence of a peri-articular soft-tissue abscess was independent of the route of infection and the advanced imaging modality (Table 2).

**Figure 2:**
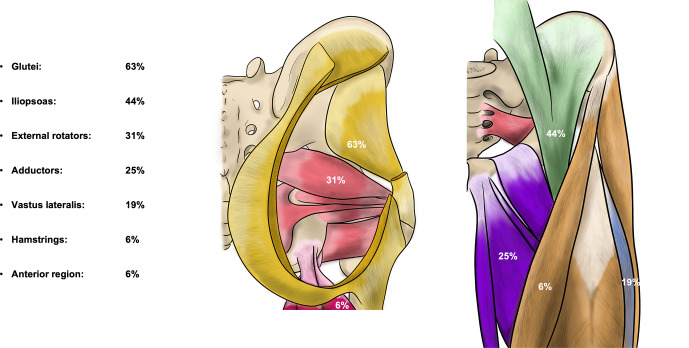
Percentage of patients with substantial abscesses specified per region. Yellow – gluteal region (gluteus maximus, medius and minimus muscle), green – iliopsoas region, red – external rotator region, purple – adductor region, pink – hamstring region, blue – vastus lateralis region, and orange – anterior region (tensor fascia latae (TFL) muscle, sartorius muscle, and/or rectus femoris muscle).

### 3.3 Patterns of infectious manifestations

Out of the 16 patients with at least one abscess, a collection was present in the gluteal region in 10 patients (63 %) and in the iliopsoas region in 7 patients (44 %). Together, these compartments were affected in 88 % of patients with at least one abscess. The other regions were less frequently involved, ranging from one to five patients (Fig. 2). All together, these other regions were affected in 56 % of patients and most often in combination with either the gluteal region, the iliopsoas region, or both (7 / 9 patients). Eight patients had a collection in only one compartment, two patients in two, and two patients in three compartments. In one patient, an aerated collection was present in all seven soft-tissue compartments.

**Figure 3:**
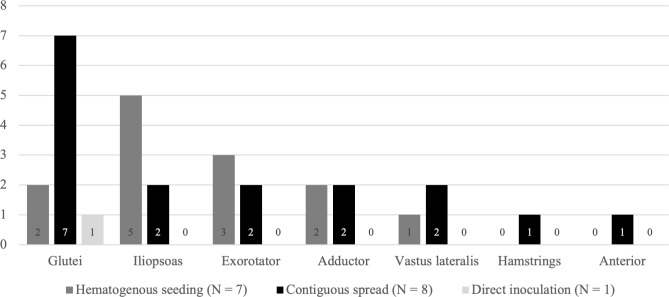
Regions involved in abscessation by route of infection.

**Figure 4:**
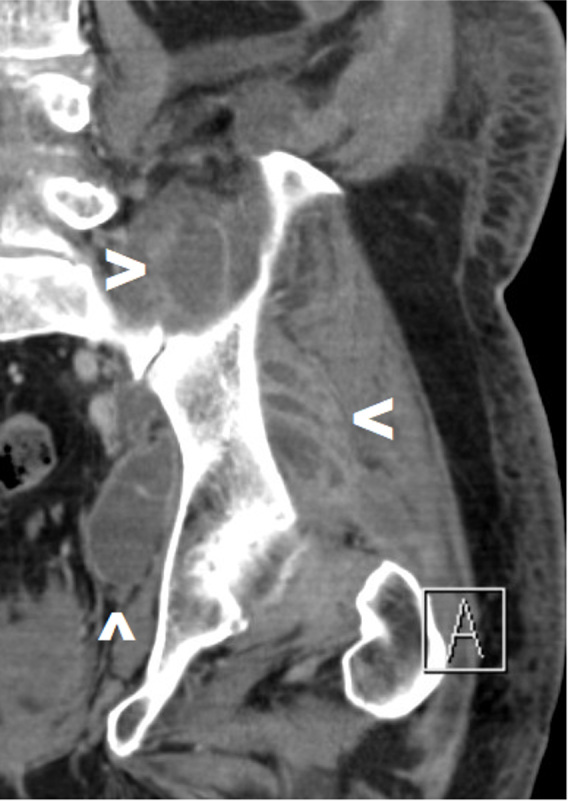
Pre-operative CT scan of a patient with SA of the left native hip due to hematogenous seeding with abscesses in the gluteal, iliopsoas, and external rotator regions (see arrows). A combination of a posterolateral approach and a Stoppa approach was deemed necessary.

When comparing patterns of infectious manifestations based on the origin of the SA, we excluded the inoculation group since only two patients had extra-articular involvement, and just one patient had a substantial collection. The number of regions with a substantial abscess in patients with SA of the native hip due to contiguous spread did not differ significantly from patients of the hematogenous group ( p=.709 ). We did, however, find a significantly higher involvement of the gluteal region in patients with SA due to contiguous dissemination compared to patients with SA due to hematogenous seeding ( p=.041 ). In contrast, the iliopsoas region was more frequently involved in patients with SA due to hematogenous seeding (5 / 7 patients in the hematogenous group and 2 / 8 patients in the contiguous spread group) (Fig. 3). However, this difference was not found to be statistically significant ( p=.132 ).

### 3.4 Surgical implications

In all patients with gluteal region involvement, a posterolateral approach of the hip joint was deemed most appropriate. Furthermore, this approach was also selected in a patient with solitary external rotator compartment involvement and in a patient with a collection extending from intra-articular into the caudal portion of the iliopsoas muscle. In some cases, with multicompartmental collections, an additional incision was proposed to provide access to a more remote soft-tissue region (lateral window of the ilioinguinal approach, Stoppa approach (Fig. 4), anterior quadriceps approach). A (extended) direct anterior approach was opted for in three patients: one patient had a solitary abscess in the adductor region, one patient in the iliopsoas, and one patient had a collection in both the adductors and the iliopsoas. A direct lateral approach was selected in one patient with both iliopsoas and vastus lateralis collections.

**Table 3:** Prognostic implications of extensiveness on advanced imaging.

	Overall	Prolonged hospital	Lack of infection	Preservation of
		stay ( >60 d)	control within ≤2	femoral head
			interventions	
Extent of infection on all imaging				
N	24	17	6	5
Number of regions with abnormalities	4.29±2.27	4.06±2.44	5±2.28	2.8±2.39
Collection present	15/24 (63 %)	11/17 (65 %)	5/6 (83 %)	2/5 (40 %)
Number of regions with a collection	1.25±1.57	1.35±1.73	2.17±2.48	0.6±0.89
Extent of infection on MRI				
N	13	6	2	3
Number of regions with abnormalities	5.62±1.66	$6.5\pm 0.84^{*}$	7±0	$3.67\pm 2.52^{*}$
Collection present	9/13 (69 %)	5/6 (83 %)	2/2 (100 %)	1/3 (33 %)
Number of regions with a collection	1.15±0.99	1.33±0.82	1.5±0.71	0.67±1.15
Extent of infection on CT				
N	16	12	6	3
Number of regions with abnormalities	2.5±1.97	2.75±1.91	3.5±2.07	1.67±2.08
Collection present	11/16 (69 %)	9/12 (75 %)	5/6 (83 %)	2/3 (67 %)
Number of regions with collection	1.88±1.93	2.17±2.04	2.67±2.5	1.33±1.53
Extent of infection on FDG-PET				
N	7	6	1	2
Number of regions with abnormalities	3±1.83	2.5±1.38	4± NaN	1.5±2.12
Collection present	3/7 (43 %)	2/6 (33 %)	0/1 (0 %)	1/2 (50 %)
Tumor to background ratio	3.94±1.56	4.06±1.67	5.52± NaN	2.51±1.68

^*^ Indicates a substantial difference lacking statistical significance.

### 3.5 Prognostic value

Prolonged hospital stay ( >60 d of hospitalisation), infection control within two surgical interventions, and preservation of the femoral head were used to evaluate the prognostic value of radiological findings. One case was excluded from prognostic analysis since this patient passed away 11 d after diagnosis due to multiple organ failure. For advanced imaging in general, as well as for each distinct type of imaging, we found no significant association between the extent of infection (i.e. the number of regions involved on imaging, the presence of a collection, the number of regions with a collection) and the outcome measures mentioned above (Table 3).

### 3.6 Differences regarding different advanced imaging modalities

In patients receiving CT imaging as the first imaging modality, a secondary, different type of investigation was conducted in 69 % of cases (Table 1). On the contrary, in patients receiving FDG PET-CT or MRI as the first type of advanced imaging, a secondary type of investigation prior to initial debridement was never conducted. Using the Fisher–Freeman–Halton exact test, this difference in reinvestigation rate between the distinct advanced imaging modalities was found to be significant ( p=.003 ). Outcome measures of patients receiving multiple types of advanced imaging interventions prior to initial debridement did not differ significantly from those of patients who did not. Furthermore, there was no significant difference in extra-articular involvement between the primary and secondary investigation.

Whole-body FDG PET-CT provides additional information (e.g. distant foci of infection) that might be of impact on therapeutic management. In our sample, other joints (mainly the shoulders) were involved in the SA in three out of seven patients receiving FDG PET-CT (in all of them, the SA was due to hematogenous seeding). No other foci of infection, such as spondylodiscitis or endocarditis, were present.

The sensitivity of MRI was significantly higher for peri-articular soft-tissue abnormalities ( p=.002 ). All advanced imaging investigations were, however, equally performant in the detection of extra-articular abscesses.

## 4 Discussion

For this study, a comprehensive analysis of advanced imaging in 25 patients with SA of the native hip joint was performed. It was found that one-third of patients had an abscess in one anatomical region, one-third of patients had abscesses in multiple anatomical regions, and only one-third of patients had no substantial abscess. Gluteal abscesses were especially common in patients with SA due to contiguous spread, while abscesses in the iliopsoas region were more common in patients with hematogenous infections. Different surgical approaches or a combination of approaches were deemed necessary to adequately deal with these various presentations of SA of the native hip joint. No significant prognostic factors could be identified.

The finding of extra-articular abscesses in 64 % of patients has several implications. Firstly, it shows that SA of the native hip might behave differently from other forms of septic arthritis, for example, the knee joint. Literature regarding the prevalence of soft-tissue abscesses in SA of native joints is scarce; however, we suspect that the frequent presence of extra-articular collections is a characteristic unique to SA of the native hip. This could be the result of the delay in presentation/diagnosis that is frequently present due to the deep location of the hip joint, the fact that it often concerns patients with underlying comorbidities (including immune deficiencies and paraplegia) (Ruythooren et al., 2023; Huang et al., 2020), and the unfamiliarity with the pathology (Ross, 2017; Kennedy et al., 2015; Carpenter et al., 2011). These factors could, in turn, explain the observation by Huang et al. (2020) that SA of the hip joint is associated with the highest risk for mortality compared to SA of other (more superficial) joints (Kao et al., 2019).

Secondly, it questions the suitability of arthroscopic debridement as a stand-alone intervention, as current guidelines on pyomyositis recommend drainage if an abscess is present (pyomyositis stage two or three) (Baddour, 2022). However, to date, direct comparison between open and arthroscopic debridement has not shown a significant difference in outcome between the two treatment modalities (Khazi et al., 2020; de Sa et al., 2015). We do note that those findings are based on retrospective studies where the initial incentive of surgeons choosing arthroscopic lavage remains unclear. It is possible that arthroscopy was mostly performed in patients with less extensive infections.

Thirdly, it poses the question of whether advanced imaging should be performed on a regular basis prior to initial debridement. After all, it is needed to determine the most efficient surgical approach since our findings showed that one standard approach does not suffice to access all the observed extra-articular lesions. In addition, it would allow for a more patient-tailored treatment protocol and could lower the number of surgical interventions needed. Elsissy et al. (2020), in contrast, suggest that advanced imaging should be used very insightfully when clinical suspicion of SA is high as it might unnecessarily delay surgical intervention; however, SA of the hip might be an exception to this rule. A large retrospective review on septic arthritis of the native joints performed by Lauper et al. (2018) did not find a significant difference in the risk of sequelae when surgical intervention is delayed in patients without sepsis. Furthermore, as noted above, a significant delay is often already present in patients with SA of the native hip. In our sample, the median delay until initial debridement was 12 d, with an interquartile range of 15. The question remains of which type of imaging would be most suited.

CT is the most readily available and widespread type of investigation in the acute setting (Eurostat, 2021). This corresponds with our findings, where two out of three patients receiving advanced imaging underwent a CT investigation. Yet, receiving pre-operative CT imaging was significantly correlated with further investigation via MRI or FDG PET-CT, independent of the presence of an abscess. This questions the value of CT imaging in the surgical management of SA of the hip, as based on our sample, it seems surgeons are not satisfied with the information provided by the investigation. There was, however, no significant difference in the outcome. It is important to note that due to the retrospective nature of this study, we cannot make any assumptions on the value of CT imaging in diagnosing patients with potential SA since our sample was restricted to patients in whom bacterial infection of the joint was confirmed.

MRI is the imaging modality of choice in musculoskeletal infections because of its superior representation of soft tissues and sensitivity for pathological oedema (Simpfendorfer, 2017). As expected, we found that the sensitivity of MRI was significantly higher for peri-articular soft-tissue abnormalities ( p=.00 2). This was, however, not the case for the detection of abscesses. We therefore suspect that the added soft-tissue resolution of MRI might not be indispensable in the management of SA of the hip. In addition, MRI is less widely available and cannot be performed in some patients (e.g. claustrophobia, implanted devices) (Simpfendorfer, 2017).

Whole-body FDG PET-CT provides additional functional information and can detect distant foci of infection (Casali et al., 2021). This is particularly helpful in patients presenting with less specific clinical findings or when multi-joint involvement is suspected. In our series, six out of seven patients who underwent FDG PET-CT had an infection due to hematogenous seeding with a less specific clinical appearance (e.g. fever of unknown origin) or pain in multiple joint regions. We found no significant difference in outcome regarding the different types of advanced imaging. We therefore conclude that any kind of pre-operative advanced imaging could suffice, and choices should be based on availability and clinical presentation. If directly available, MRI is the investigation of choice in patients with overt and exclusive involvement of the hip joint and FDG PET-CT in patients where the clinical focus is less uniform or hematogenous spread is expected. These examinations provide more satisfactory information for surgical planning and thus may avoid the need for a second investigation.

This study has several limitations, the most important being its small sample size. Despite including all patients treated during a 16-year period at a large university hospital ( ±2000 beds), only 41 patients with SA of the native hip joint were eligible for inclusion, and only 25 received some form of advanced imaging pre-operatively. This could be a rationale for not finding significant prognostic factors associated with a prolonged hospital stay, infection control within two interventions or fewer, and preservation of the femoral head. Therefore, we believe that – at present – treatment decisions should be mainly based on the route of infection and the state of the joint, as previously suggested (Ruythooren et al., 2023). The extent of extra-articular abscesses should guide the surgical approach but not the decision on whether to preserve, sacrifice, or replace the hip joint. A second limitation is that imaging did not happen using a single standardised protocol and was performed in multiple different centres utilising diverse equipment. Furthermore, selection bias is present since not every adult patient with expected SA of the native hip received advanced imaging, and no standardised protocol was in place to determine who did. Lastly, our sample might not be directly extrapolatable to other centres or countries.

## 5 Conclusions

Based on the results of this study, we recommend performing advanced imaging in patients with suspected or proven septic arthritis of the native hip joint, as abscesses are present in 64 % and might require varying anatomical approaches.

## Data Availability

Raw and anonymized data are available for perusal at https://doi.org/10.17632/4nvsv9jdgt.1 (Cools, 2024).
